# A Case of Radiologically Compatible Chronic Lymphocytic Inflammation With Pontine Perivascular Enhancement Responsive to Steroids (CLIPPERS) With Demyelinating Lesions

**DOI:** 10.7759/cureus.43000

**Published:** 2023-08-05

**Authors:** John M Coggins, Ahmed Harazeen, Rasha Alfattal, Kassandra Corona, Peeyush Bhargava, Michelle Felicella, Xiangping Li

**Affiliations:** 1 Neurology, University of Texas Medical Branch at Galveston, Galveston, USA; 2 Pathology, University of Texas Medical Branch at Galveston, Galveston, USA; 3 Radiology, University of Texas Medical Branch at Galveston, Galveston, USA

**Keywords:** histopathology, myelin-oligodendrocyte glycoprotein, tuberculosis, demyelination, clippers syndrome

## Abstract

Chronic lymphocytic inflammation with pontine perivascular enhancement responsive to steroids (CLIPPERS) is a recently identified diagnosis that can cause a variety of severe symptoms, including ataxia, dysarthria, diplopia, paraparesis, and vertigo. These symptoms rarely present in isolation but often accompany one another in various combinations. Magnetic Resonance Imaging (MRI) of the brain is critical for making the diagnosis and typically reveals scattered enhancement within the pons and adjacent structures. The syndrome responds well to high-dose steroids, and maintenance therapy is required to prevent a recurrence. In this report, we present a case of a 62-year-old man who developed CLIPPERS syndrome. The patient presented with hemiparesis and dysarthria, which developed over four months and then acutely worsened within 24 hours. After diagnosing CLIPPERS, the patient was placed on high-dose steroids and experienced rapid clinical improvement, as well as improvement on repeat MRI. The patient’s treatment was complicated by an incidental diagnosis of tuberculosis, which required simultaneous management with isoniazid.

## Introduction

Chronic lymphocytic inflammation with pontine perivascular enhancement responsive to steroids (CLIPPERS) is a syndrome first described in 2010 [1]. Four core features define the diagnosis: subacute neurological complaints (most commonly ataxia, dysarthria, and diplopia), numerous enhancing lesions bilaterally “peppering” in two of the three following structures: pons, brachium pontis, and cerebellum, a robust response to steroid therapy, and CD4+ T-cell infiltrate on histopathology [2]. Alternative diagnoses should also be thoroughly excluded. The mean age at diagnosis is 46, and the most commonly reported symptom is ataxia [3]. Rapid initiation and continued maintenance of steroid therapy are necessary to prevent relapses. High-dose steroids are recommended during the acute phase for at least 10 days, and azathioprine is the most commonly utilized steroid-sparing maintenance therapy [3].

The pathophysiology of CLIPPERS remains largely unknown. The researchers who originally described the syndrome hypothesized an immune-mediated attack on unknown epitopes in perivascular regions could explain the findings [1]. Others speculate that an exogenous trigger may prompt the immune reaction, particularly in two cases of CLIPPERS following vaccine administration [4]. This proposed exogenous trigger is supported by the predominance of CD4+ T-cells on histopathology, which respond to MHC-II proteins typically carrying exogenous proteins. Recent correlation with anti-myelin-oligodendrocyte glycoprotein antibodies (MOGAb) could indicate an etiology more akin to demyelinating disease [5]. Understanding the pathophysiology of CLIPPERS syndrome in greater detail could greatly benefit the diagnostic process and guide future refinement of treatment strategies.

## Case presentation

**Figure 1:**
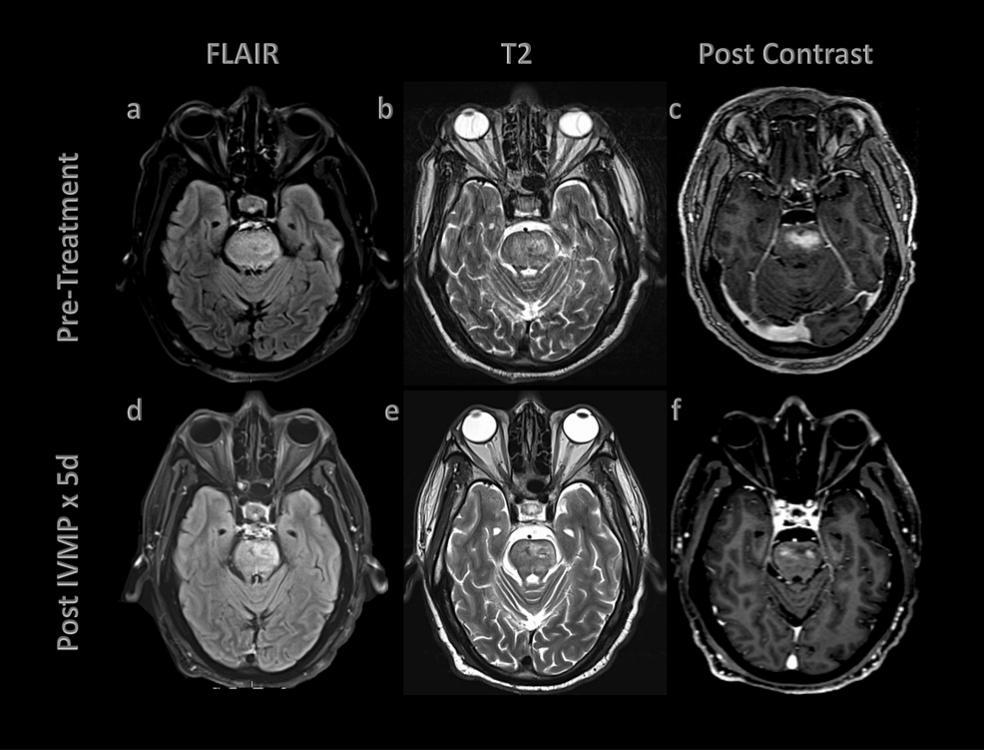
Before and after response to steroids in CLIPPERS patient as demonstrated on axial postcontrast T1-weighted MRIs (a) Axial fluid-attenuated inversion recovery (FLAIR) image shows expansile hyperintense lesion involving the pons. (b) Axial T2-weighted image shows intermediate signal intensity in the pons. (c) Axial postcontrast T1-weighted image shows enhancing lesion involving the pons. (d) FLAIR imaging shows a slight reduction of hyperintense lesions compared to prior imaging. (e) Axial T2-weighted image shows unchanged intermediate signal intensity in the pons. (f) Five days after treatment with IVMP, axial postcontrast T1-weighted image shows reduced enhancement of the pons. CLIPPERS - Chronic lymphocytic inflammation with pontine perivascular enhancement responsive to steroids

A 62-year-old male with a past medical history of type 2 diabetes mellitus, hyperlipidemia, and hypertension presented with progressive right-sided weakness over the past four months. The patient’s weakness was slowly progressing and eventually required him to use a cane to ambulate. He had fallen on several occasions and was dragging his right foot. One day prior to arrival, his weakness rapidly worsened and was accompanied by the development of slurred speech. On presentation, he was unable to raise his right arm or right leg. His ability to give a detailed history was severely limited by his dysarthria. He denied vision changes, numbness, vertigo, word-finding difficulty, or ataxia. On physical exam, the patient showed signs of dysarthria without facial droop. Strength in the right extremities was 3/5 for all movements and 5/5 in the left extremities. A computed tomography (CT) head without contrast was obtained, showing hypodensity of the midbrain and pons. CT angiogram of the head and neck showed chronic left vertebral artery occlusion with no acute findings. Two days following admission, the patient had not improved, and a brain magnetic resonance imaging (MRI) was obtained, which showed a T2/FLAIR hyperintense lesion with multiple punctate and patchy contrast enhancement involving the pons and to a lesser extent the midbrain and medulla (Figures 1a-1f). Cerebrospinal fluid (CSF) was remarkable for elevated protein (141 mg/dL) and elevated white blood cell count (WBC) (46 cells/µL). CSF meningitis panel and culture were negative. CSF oligoclonal blands showed zero bands.

**Figure 2:**
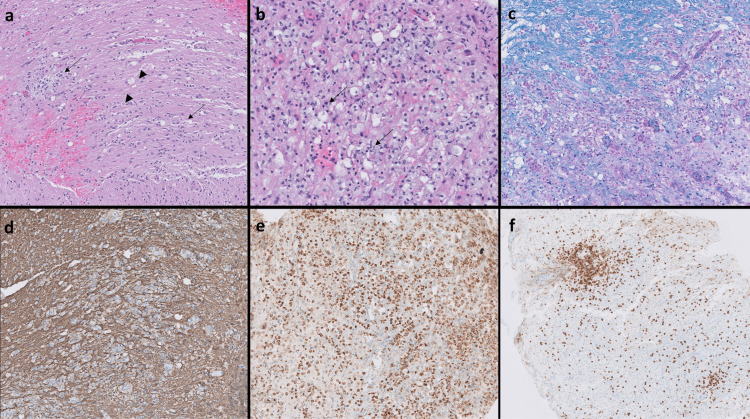
Histopathologic features of pontine biopsy (a, b) Low- and high-power micrographs of hematoxylin and eosin (H&E)-stained sections (50x and 200x, respectively) demonstrate pontine parenchyma with multiple foci with prominent foamy macrophage infiltrate (arrows) and scattered axonal spheroids (arrowheads, 200x) representing some axonal damage. (c) Luxol Fast Blue and Periodic Acid–Schiff (LFB-PAS) stain reveals prominent myelin loss (right lower) in areas of macrophage infiltrate (LFB-PAS, 100x), with relative preservation of the axons confirmed by neurofilament stain (d) (Neurofilament, 100x). (e) CD68 highlights the dense macrophage infiltrate (CD68, 100x). (f) CD3 shows T-cell predominant, lymphocytic perivascular infiltration with less dense parenchymal infiltrate (CD3, 50x).

A stereotactic biopsy of the pons was performed, and the histopathologic features are illustrated in Figures 2a-2f. The biopsy revealed pontine parenchyma with macrophage infiltrate, myelin loss, and gliosis, most consistent with active inflammatory demyelinating disease. There are areas of prominent myelin loss, which is highlighted on Luxol Fast Blue/PAS stain. There was no evidence of vascular damage, vasculitis, granulomas, or a neoplastic process. No viral cytopathic effect was seen and immunohistochemistry for SV-40, HSV-I, HSV-II, and CMV were negative. Special stains were negative for fungal and acid-fast microorganisms.

Given these results, the patient was placed on 1g intravenous methylprednisolone (IVMP) for five days. During workup, the patient tested positive for tuberculous using Quantiferon Gold, causing concern that the needed high-dose steroids would reactivate the tuberculosis. With infectious disease consultation, a decision was made to continue with high-dose steroids in conjunction with isoniazid therapy. The patient saw great improvement in symptoms and diminished findings on repeat MRI brain. Following the five days of IVMP, the patient was placed on a five-month prednisone taper to prevent relapse of CLIPPERS syndrome. At two months follow-up, his right-sided weakness had greatly improved; however, he continued to drag his right leg, for which he was receiving physical therapy. Maintenance steroid therapy was not prescribed following completion of prednisone taper due to poorly controlled diabetes, continued management of latent tuberculosis, and resolution of CLIPPERS symptoms.

## Discussion

The presentation of steroid-responsive brainstem inflammation, characteristic radiological findings of brainstem punctate enhancements, CSF lymphocytic leukocytosis, and brain biopsy findings are suggestive of CLIPPERS syndrome in our case. The patient’s symptoms progressed over the course of four months and then acutely worsened over 24 hours, consistent with typical CLIPPERS syndrome chronicity, which is subacute with episodic exacerbations [6]. While a wide variety of clinical presentations are possible, the most common symptoms are ataxia and diplopia [2]. In our case, the patient developed dysarthria and hemiparesis. A review of 60 previously reported cases of CLIPPERS syndrome showed that dysarthria and some forms of paresis were present in 63% and 35% of patients with CLIPPERS, respectively [7]. The most common symptom was ataxia which was present in 97% of patients; this patient denied symptoms of ataxia [7].

Since the initial differential diagnoses also included an underlying infectious process (rhombencephalitis), inflammation/vasculitis, autoimmune disease, or low-grade neoplasm, a brain biopsy was performed to exclude other processes, in particular, central nervous system (CNS) lymphoma. Biopsies are not required for the diagnosis of CLIPPERS syndrome but are helpful in the exclusion of alternative pathologies [8]. In our patient, a biopsy of the pons revealed T-cells in perivascular foci, as well as signs of an active inflammatory demyelinating process. The presence of demyelinating lesions and perivascular T-cells is an atypical finding for CLIPPERS and might suggest a possible pathophysiological connection between CLIPPERS and demyelinating diseases. This connection is further supported by a previously proposed relationship between CLIPPERS and anti-MOGAb [5]. MOGAb have received increasing attention in recent years, and although our study is limited by a lack of testing for anti-MOG, the brain biopsy result of active inflammatory demyelinating processes contributes to the idea that a pathophysiological connection between CLIPPERS and demyelinating diseases may exist and contribute to the disease pathology.

There are two limitations regarding our case. First, the serum and CSF autoimmune neurologic disease panel were not sent. Second, there was no long-term follow-up with the neurology clinic to better define this clinical syndrome. The treatment of this patient’s CLIPPERS was also complicated by an incidental diagnosis of latent tuberculosis. After discussion with infectious disease, the patient was placed on isoniazid therapy concomitant with high dose methylprednisolone. The patient recovered well with no reactivation of tuberculosis. Mele et al. document a related case in which a patient was solely treated for tuberculosis before the diagnosis of CLIPPERS was fully realized [9]. The patient’s neurological symptoms were initially suspected to be caused by CNS tuberculosis due to a positive Qauntiferon Gold test (QFT-G), however, was later determined to not possess an active infection. Interestingly, the patient’s neurological symptoms diminished during rifampin, isoniazid, and pyrazinamide therapy and only relapsed after the therapy was withdrawn. They speculated that the anti-inflammatory effects of rifampin could have provided modest relief from the CLIPPERS symptoms and prevented it from relapsing. The patient in this case is currently being treated with only isoniazid therapy after a five-month prednisone taper due to type 2 diabetes mellitus and concerns about tuberculosis reactivation. The patient has not reported any relapse in symptoms, which could be related to a similar anti-inflammatory effect of isoniazid [10].

## Conclusions

This report of a new diagnosis of CLIPPERS syndrome demonstrates the characteristic clinical course, MRI findings, and responsiveness to steroids, along with histological features of demyelination and perivascular lymphocytic infiltrate. It highlights the variety of clinical presentations with CLIPPERS syndrome and its possible pathophysiological connection with a demyelinating process. The patient’s symptoms of hemiparesis and dysarthria comprised a less common presentation. The incidental positive tuberculosis test complicated the use of high-dose steroids but did not preclude recovery. Much work remains to be done regarding the pathophysiology and treatment options in this intriguing disease entity.
